# Evaluation of the Mechanism of Modified Lingguizhugan Decoction in the Treatment of Nonalcoholic Fatty Liver Disease

**DOI:** 10.1155/2022/4576679

**Published:** 2022-01-25

**Authors:** Fuyuan Yang, Jianxiang Zhou, Bo Su, Qiong Zhang, Xuezhi Luo, Fei Wang, Jiangrong Huang, Wei Huang

**Affiliations:** ^1^School of Basic Medicine, Yangtze University Health Science Center, Jingzhou, Hubei 434020, China; ^2^Department of Endocrinology, Jingzhou Hospital of Traditional Chinese Medicine, Third Clinical Medical College, Yangtze University, Jingzhou, Hubei 434020, China; ^3^School of Basic Medicine, Yangtze University Health Science Center, Jingzhou, Hubei 434020, China; ^4^Hubei College of Chinese Medicine, Jingzhou, Hubei 434020, China

## Abstract

Nonalcoholic steatohepatitis (NASH) may develop into cirrhosis and liver cancer, which imposes a great burden to individuals and society. Lingguizhugan decoction is a commonly used dampness dispelling medication in traditional Chinese medicine and is often used to treat those with phlegm and retained fluid from various causes and pathogeneses. The objective of this study was to explore the effect and mechanism of modified Lingguizhugan decoction (MLGZG) on lipid metabolism and the inflammatory response to identify a theoretical basis to promote its clinical application in NASH therapy. After treatment with MLGZG for 8 weeks, the weight of high-fat-diet (HFD)-fed NASH rats was significantly higher than that of rats in the normal group, and the weights in each dose group were significantly lower than those in the model group. The treatment groups (low, medium, and high doses) had different degrees of improvement in the changes in hepatocyte tissue structure, steatosis, and inflammatory infiltration. Compared with that in the normal group, the expression of TNF receptor-associated factor-3 (TRAF-3) and nuclear factor *κ*B (NF*κ*B) in the model group significantly increased to varying degrees. Compared with the NASH group, the treatment groups (low, middle, and high doses) showed modified lipid metabolism gene expression and decreased inflammatory factor expression levels. Modified Lingguizhugan decoction can improve the general condition of rats with nonalcoholic fatty liver disease by reducing the expression levels of TRAF3, NF-*κ*B, the Toll-like receptor 4 (TLR-4) pathway, and related proteins, as well as the expression levels of lipid metabolism genes and cytokines.

## 1. Introduction

Nonalcoholic steatohepatitis (NASH) is the exacerbated form of nonalcoholic fatty liver disease. While the etiology and pathogenic mechanisms underlying NASH remain largely elusive, the development of NASH results from hepatocyte damage, inflammation, and tissue destruction in the liver, leading to metabolic system and circulatory system dysfunction. Thus, NASH is a great threat to human health. Despite enormous efforts to optimize existing drugs and develop new drugs in recent years, a large portion of NASH patients are still not responsive to medical treatment. The chronic inflammatory response has been shown to contribute to the development of NASH. Genetic factors and abnormal nutritional metabolism lead to the accumulation of adipose tissue in obese patients. Toll-like receptor 4 (TLR-4) can be activated by saturated fatty acids and then upregulates the release of proinflammatory factors such as tumor necrosis factor-*α* (TNF-*α*), interleukin-6 (IL-6), and monocyte chemoattractant protein-1 (MCP-1), leading to the occurrence of a chronic inflammatory response [1–3]. Then, insulin resistance is caused by nuclear factor *κ*B (NF-*κ*B) and C-Jun N-terminal kinase (JNK) [4]. The accumulation of CD8 T cells mediated by inflammatory factors in obese patients enhances insulin resistance through the mislocation of glucose transporter type 2 (GLUT-2) [5]. Furthermore, insulin resistance and increased circulating-free fatty acids cause intrahepatic fat accumulation, which, combined with chronic inflammatory response-induced cytotoxicity, results in damage to liver cells and an overactive inflammatory response [6].

In multiple cell types, including hepatocytes, cardiomyocytes, osteoclasts, microglia, and inflammatory cells, TRAF3 is expressed and plays a key role in the regulation of inflammation [7–10]. Animal and clinical evidence indicates that the function and expression of TRAF3 are involved in the pathogenesis of a variety of inflammation-related diseases, such as inflammatory bowel disease, liver inflammation, diabetes, bone inflammation, encephalitis, and autoimmune inflammation. TRAF3 serves as an E3 ubiquitination signal transducer in inflammatory responses and is activated by TNF receptor (TNF-R) superfamily receptors, Toll-like receptors (TLRs), NOD-like receptors (NLRs), retinoic acid-inducible gene I (RIG-I), and cytokines [11,12]. TRAF3 expression is significantly increased in the liver of obese mice, and hepatocyte-specific TRAF3 knockout can alleviate insulin resistance. TRAF3 leads to transforming growth factor beta-activated kinase 1 (TAK1) ubiquitination and autophosphorylation in high-fat diet-fed mice and then activates the NF-*κ*B inflammatory pathway and JNK-IRS1 (insulin receptor substrates 1) insulin resistance [13].

The traditional classic prescription Lingguizhugan decoction, originally recorded in Shanghan Zabing Lun, has been commonly used for the treatment of dizziness and palpitations. Based on years of clinical NASH treatment experience, we previously formulated a modified Lingguizhugan decoction (MLGZG) by adding herbs to the original prescription and achieved satisfactory efficacy in the treatment of obesity, insulin resistance, and other metabolic diseases, but the underlying mechanism of this effect is still unclear [14]. Modified Lingguizhugan decoction has many advantages in metabolic disease therapy [15]. It has been reported that Lingguizhugan decoction could inhibit TNF-*α* synthesis in the treatment of chronic heart failure. In NASH patients, the expression of the TNF-*α* gene and the inflammatory response were effectively reduced by Lingguizhugan decoction [16]. Our previous animal study demonstrated that MLGZG reduced blood glucose, fat, body weight, and insulin resistance in HFD rats. In addition, MLGZG was able to ameliorate obesity in rats with metabolic syndrome and reduce fat cell volume in obese rats [17]. Given that therapeutic strategies for NASH largely contribute to immune and inflammation modulation, we undertook the present study to check whether MLGZG can regulate inflammatory reactions through animal experiments and to explore the signaling pathways underlying the effect. After administration of MLGZG to an HFD-fed rat model of NASH, we detected the effects on serum lipids, inflammation levels, and the TNF*α*-TRAF3-related signaling regulation pathway in model animals. MLGZG may serve as a novel mechanism for the treatment of NASH, as decreasing bile acid (BA) and farnesoid *X* receptor (FXR) signaling may yield metabolic benefits [8, 14–19].

## 2. Materials and Methods

### 2.1. Preparation of MLGZG

MLGZG is composed of 12 grams (g) Cortex Poria, 9 g Ramulus Cinnamomi, 12 g Rhizoma Atractylodis Macrocephalae, 12 g Radix Codonopsis, 12 g Rhizoma Pinelliae, 30 g Fructus Crataegi, 15 g Herba Siegesbeckiae, 9 g Carthamus tinctorius, 9 g Ligusticum wallichii, 15 g Radix Polygonum Multiflorum Preparata, and 6 g honey-fried licorice root. These components were soaked in 1125 ml distilled water for 30 min, followed by 40 min of boiling at 100 °C. Then, 846 ml distilled water was added and boiled for 30 min, the liquid was collected, and the two doses were mixed. The decoction was then condensed to 1 ml and stored at 4 °C. Before use, it was warmed to 37 °C before use.

### 2.2. Quantitative Real-Time (qRT) PCR

Total RNA was isolated using TRIzol Reagent (Invitrogen, Waltham, MA, USA). For reverse transcription, 1–5 mg total RNA was used, following the methods of Yang et al., 2018 [20]. First-strand complementary DNA synthesis was performed using oligo(T)18 and M-MLV enzyme (Promega, Madison, WI, USA). A PCR system was applied to amplify target genes (thermal cycling program: 1 : 94 °C 4 minutes (min); 2 : 94 °C 30 seconds (s); 3 : 55 °C 30 s; 5 : 72 °C 30 s; and 4 : 72 °C 10 min; steps 2–5 were repeated for 30–35 cycles). The mRNA levels were measured by real-time PCR (Applied Biosystems) using SYBR Green Master Mix (Applied Biosystems). The total amount of mRNA was normalized to endogenous glyceraldehyde 3-phosphate dehydrogenase mRNA. The sequences of the primers are shown in Table 1.

### 2.3. Western Blot

Tissues were lysed at 4°C using radioimmunoprecipitation assay lysis buffer (RIPA, Thermo Fisher, Waltham, MA, USA) with a protease inhibitor cocktail for 30 min. Supernatants were collected, and the protein concentrations were measured by a Bradford protein assay system (Bio-Rad, Hercules, CA, USA). Proteins were incubated with primary antibodies overnight at 4°C, washed and incubated with horseradish peroxidase-conjugated secondary antibodies diluted 1 : 10,000 in 2% milk in Tris-buffered saline Tween-20. Antibodies used in the Western blot are listed in Table 2. The membranes were washed, and protein bands were detected with enhanced chemiluminescence reagent (Amersham, Little Chalfont, UK) and a ChemDoc^TM^ XRS + system (Bio-Rad, Hercules, CA, USA) [21]. For quantification, densitometry in ImageJ was applied to quantify the relative band intensities.

### 2.4. HFD NASH Rat Model

Mature male Wistar rats (weight 300–350 g, purchased from Hubei Provincial Laboratory Animal Center) were used in this study. All animal experiments were approved by the Institutional Animal Care and Use Committee of Yangtze University (CJYXBEC2018-113, 2018.11.11) and were conducted in accordance with the NIH guidelines and regulations on animal experimentation. Rats were fed a high-fat diet (fat provides 60% of the total energy) for 12 consecutive weeks to establish the model. The weights of the rats were measured every 2 days throughout the whole experiment [22]. Rats were randomly divided into five groups: a blank group, a NASH group, an MLGZG low-dose group, an MLGZG moderate-dose group, and an MLGZG high-dose group. MLGZG (6 g/(kg•day), 12 g/(kg•day), and 18 g/(kg•day)) was gavaged to rats in the MLGZG low-dose, MLGZG moderate-dose, and MLGZG high-dose groups. Rats in the control and NASH groups were gavaged with saline. At different time points, rats were sacrificed, and their livers were collected for further studies.

### 2.5. Hematoxylin and Eosin (H&E) Staining

Paraffin-embedded colons were prepared in 6-*μ*m sections, deparaffinized using xylene and rehydrated through gradient alcohol (100% ^*∗*^2, 95% ^*∗*^2, 80% ^*∗*^1, 70% ^*∗*^1, water-1, 90 s per step with shaking). H&E staining was performed as previously described [20]. After rehydration, sections were stained with hematoxylin for 5 min and differentiated in acid ethanol (0.36 g HCl in 100 ml ethanol, pH = 1) for 3 min. Then, the sections were washed in running tap water for 2 min, blued in tap water for 10 min, and stained in eosin Y solution for 1–2 min. Then, 95% ^*∗*^2 plus 100% ∗2 alcohol was applied for dehydration, after which the specimens were cleared with xylene twice and finally mounted with histological mounting medium.

### 2.6. HFD Cell Model ELISA

After 7 days of housing and feeding, 24 male Sprague Dawley rats were randomly divided into 4 groups (Normal, MLGZG^L^, MLGZG^M^, and MLGZG^H^; 6 for each group), and saline or MLGZG was gavaged into the rats twice a day for 7 consecutive days. Then, drug-containing serum was collected as described in a previous study. Briefly, blood was collected from the abdominal aorta, quiesced for 1 h, and centrifuged at 3000 RPM for 15 min. The serum was collected and inactivated at 56 °C for 30 min and stored at -80 °C for future use [23]. HEPG2 cells were cultured in 37 °C incubators with 5% CO_2_ and DMEM containing 10% FBS and 1% penicillamine. After 12 h of starvation with DMEM without serum, the HEPG2 cells were cultured with 0.1 mM palmitic acid in complete culture medium and cultured for 24 h to establish the HFD cell model. After that, the cells were treated with 10% normal rat serum or drug-containing serum, and the cell supernatant was collected 24 h later for ELISA tests. The levels of TNF-*α*, IL-1*β*, and IL-2 in the supernatant were tested with ELISA kits from Shanghai Jing Anti-Biology Company.

### 2.7. Serum Lipid Analysis

After feeding for 12 weeks, rats from the control, HFD, and MLGZG treatment groups were subjected to blood biochemical analysis. An automated biochemical analyzer (Type AU5421, Olympus, Japan) was used to estimate the concentrations of triglycerides (TGs), total cholesterol (TC), low-density lipoprotein cholesterol (LDL-C), and high-density lipoprotein cholesterol (HDL-C) in plasma [24].

### 2.8. Statistical Analysis

All experiments were repeated at least three times, and the data are presented as the mean ± SEM. Student's *t*-test was applied to calculate the differences between two groups, while one-way analysis of variance (ANOVA) was applied for comparisons among three groups, and post hoc analyses were performed with the Newman–Keuls multiple comparison test. Statistical significance was considered at *P* < 0.05 (# indicates *P* < 0.05, ## indicates *P* < 0.01, and ### indicates *P* < 0.001 HFD group compared to the normal control group; ^*∗*^ indicates *P* < 0.05, ^*∗*^^*∗*^ indicates *P* < 0.01, and ^*∗∗∗*^ indicates *P* < 0.001 MLGZG-treated groups compared to HFD group). Statistical calculations were performed with GraphPad Prism software.

## 3. Results

### 3.1. MLGZG Ameliorates NASH in a HFD NASH Rat Model

**Figure 1:**
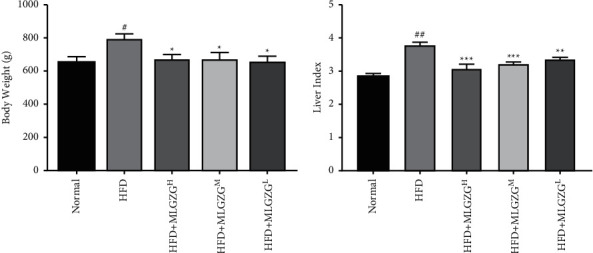
Final body mass of the rats at week 12 (A) and liver index of the rats at week 12 (B). (HFD: high-fat diet; MLGZG^L^: MLGZG low dose; MLGZG^M^: MLGZG moderate dose; MLGZG^H^: MLGZG high dose. # indicates *P* < 0.05, ## indicates *P* < 0.01, and ### indicates *P* < 0.001, HFD group compared to the normal control group; ^*∗*^indicates *P* < 0.05, ^*∗∗*^indicates, *P* < 0.01 and ^*∗∗∗*^indicates *P* < 0.001, MLGZG-treated groups compared to the HFD group).

**Figure 2:**
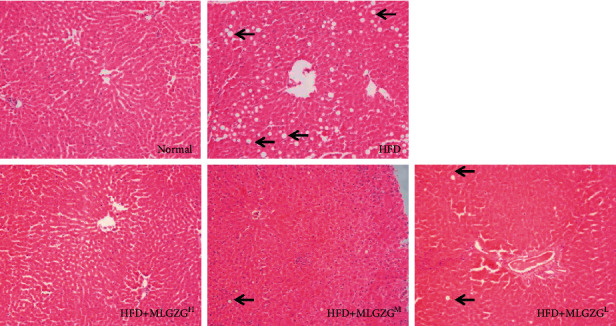
MLGZG ameliorates NASH in an HFD NASH rat model. Photographs of H&E-stained paraffin sections of livers from rats treated with saline or MLGZG (100×) (black arrow indicated hepatocyte vacuolization; HFD: high-fat diet; MLGZG^L^: MLGZG low dose; MLGZG^M^: MLGZG moderate dose; MLGZG^H^: MLGZG high dose).

In this study, to test the potential therapeutic effect of MLGZG on NASH, we used an HFD-induced NASH rat model. In this model, fed on a high-fat diet, rats presented the most evident clinical signs of NASH from week 5 to week 12, along with obvious abdominal obesity, significant weight gain in the early stage, and a tendency for weight decline in the later stage. MLGZG demonstrated a more pronounced effect on the reduction in weight at week 12 (Figure 1(a)). Meanwhile, the liver mass of HFD rats was significantly increased relative to that of normal rats, and MLGZG feeding effectively reduced the liver mass (Figure 1(b)). In MLGZG-fed HFD rats, the liver index was also significantly reduced compared with that in HFD rats. Histopathological examination of the liver sections showed that MLGZG significantly reduced the extension and severity of the inflamed area and the damage to hepatocytes compared with that of HFD-only rats. H&E staining indicated that MLGZG effectively reduced hepatic lobule structure damage, disordered cell arrangement, and the number of lipid vesicles (Figure 2). These results suggest that MLGZG administration reduces NASH development and liver damage.

### 3.2. MLGZG Modulates Blood Lipids through Lipid Metabolism Genes

**Figure 3:**
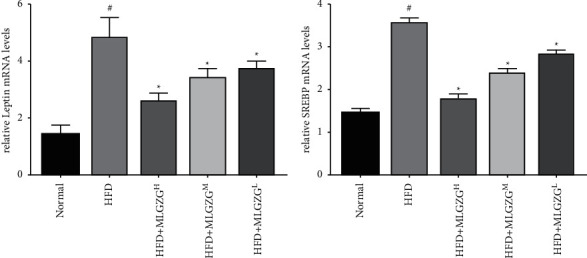
MLGZG inhibits the expression of lipid metabolic genes. Real-time PCR was used to determine the expression levels of lipid metabolic genes (a) leptin and (b) SREBP (HFD: high-fat diet; MLGZG^L^: MLGZG low dose; MLGZG^M^: MLGZG moderate dose; MLGZG^H^: MLGZG high dose; # indicates *P* < 0.05, HFD group compared to the normal control group; ^*∗*^indicates *P* < 0.05, MLGZG-treated groups compared to the HFD group).

The blood lipid content was detected, and HFD rats had a marked increase in blood lipid content relative to the control group (Table 3). Except for TG content, the TC and LDL-C contents were largely increased in HFD rats relative to control rats, and treatment with MLGZG significantly decreased the TG, TC, and LDL contents. To further characterize the therapeutic identities of MLGZG, we used real-time PCR. As shown in Figure 3, lipid metabolism genes were differentially expressed between MLGZG-fed NASH rats and NASH rats. The expression levels of SREBP and leptin were significantly downregulated by MLGZG feeding, indicating that MLGZG alters the lipid genesis of NASH rats (Figure 3).

### 3.3. MLGZG Suppresses the Immune Response during NASH

**Figure 4:**
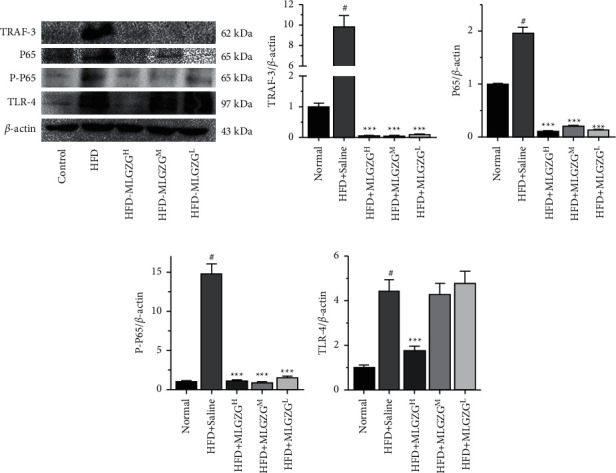
MLGZG inhibits the TLR-4/TRAF-3/NF-*κ*B pathway in NASH livers. (a) Western blot analysis was conducted using whole tissue lysates from HFD-fed NASH rat livers treated with saline/MLGZG. (b–e) Bar charts represent TRAF-3/P65/P–P65/TLR-4 expression normalized against *β*-actin. ^*∗*^*P* < 0.05 versus saline (HFD: high-fat diet; MLGZG^L^: MLGZG low dose; MLGZG^M^: MLGZG moderate dose; MLGZG^H^: MLGZG high dose; # indicates *P* < 0.05, HFD group compared to the normal control group; ^*∗∗∗*^indicates *P* < 0.001, MLGZG-treated groups compared to the HFD group).

**Figure 5:**
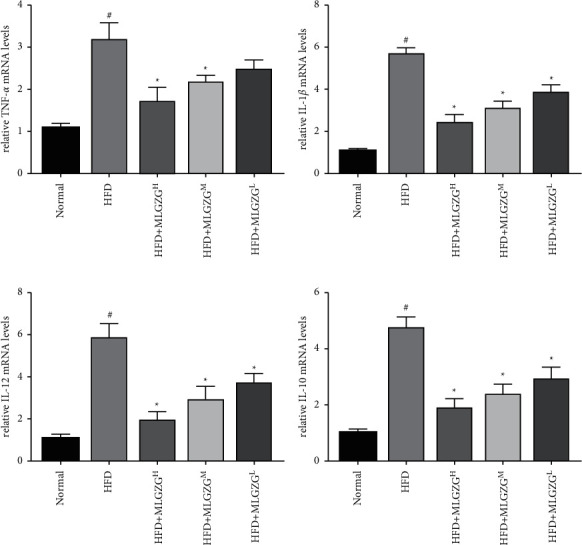
Real-time PCR was used to determine the expression levels of cytokines: (a) TNF-*α*; (b) IL-1*β*; (c) IL-12; and (d) IL-10 (HFD: high-fat diet; MLGZG^L^: MLGZG low dose; MLGZG^M^: MLGZG moderate dose; MLGZG^H^: MLGZG high dose; # indicates *P* < 0.05, HFD group compared to the normal control group; ^*∗*^indicates *P* < 0.05, MLGZG-treated groups compared to the HFD group).

**Figure 6:**
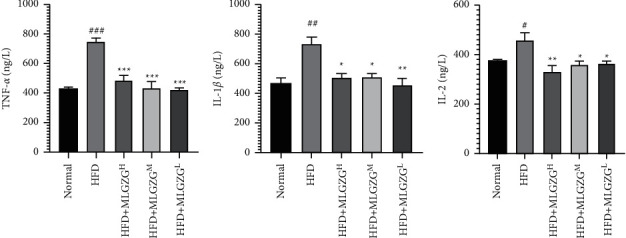
Effect of different doses of MLGZG on HFD HEPG2 cytokine secretion. TNF-*α*, IL-1*β*, and IL-2 levels in culture medium were measured by ELISA (a) TNF-*α*; (b) IL-1*β*; and (c) IL-2 (HFD: high-fat diet; MLGZG^L^: MLGZG low dose; MLGZG^M^: MLGZG moderate dose; MLGZG^H^: MLGZG high dose; # indicates *P* < 0.05, ## indicates *P* < 0.01, and ### indicates *P* < 0.001, HFD group compared to the normal control group; ^*∗*^indicates *P* < 0.05, ^*∗∗*^indicates *P* < 0.01, and ^*∗∗∗*^indicates *P* < 0.001, MLGZG-treated groups compared to the HFD group).

Interestingly, our Western blot results revealed that compared with that of HFD rats, the expression of NF-*κ*B P-p65 was significantly decreased in MLGZG-treated rats, and we also found that TLR-4 and TRAF3 expression levels were significantly reduced by MLGZG feeding. Since activation of the TLR-4/TRAF3/NF-*κ*B pathway plays a critical role in the hepatic inflammatory response, it is plausible that the therapeutic effect of MLGZG is mediated by the TLR-4/TRAF3/NF-*κ*B pathway (Figure 4). We also determined whether MLGZG was able to modulate the hepatic inflammatory reaction. We showed that MLGZG dramatically suppressed the mRNA expression of proinflammatory cytokines, such as TNF-*α* and IL-12. Since IL-10 is an anti-inflammatory cytokine and immunosuppressive factor, we also determined the effect of MLGZG on IL-10 expression. Interestingly, MLGZG significantly decreased the mRNA expression of IL-10 in HFD rat livers (Figure 5). To check the inflammatory regulatory function of MLGZG in vitro, we treated HFD HEPG2 cells with drug-containing serum. The ELISA results showed that the cytokine level in culture medium was increased by palmitic acid, and MLGZG-containing serum significantly reduced TNF-*α*, IL-1*β*, and IL-2 secretion by HFD HEPG2 cells (Figure 6).

## 4. Discussion

MLGZG is a well-established Chinese medicine prescription. This is the first study to explore the mechanism of action of MLGZG in NASH from the perspective of TRAF3-related pathways, which can provide an experimental basis for the development of an MLGZG-based NASH treatment. HFD-induced NASH is the most commonly used animal model for the study of human NASH [25]. In our study, by orally feeding rats a high-fat diet for 12 weeks, we successfully established a NASH rat model that recapitulates the early, middle, and late stages of NASH pathology. With respect to the NASH group, MLGZG appeared to offer evident therapeutic advantages in NASH treatment. First, MLGZG led to a prominent reduction in body weight (Figure 1). Second, MLGZG resulted in profound improvements in HFD-induced NASH pathology; for example, hepatocyte damage and the inflammatory response were significantly alleviated in MLGZG-treated rat livers (Figure 2). Third, the expression levels of the lipid genesis genes SREBP and leptin were reduced in MLGZG-treated livers (Figure 3), indicating that MLGZG is effective in protecting lipid metabolism function. Collectively, our results demonstrate that modified Lingguizhugan decoction is effective in reducing the clinical and pathological signs of NASH.

Although the mechanisms by which Lingguizhugan decoction regulates the immune response during the pathological processes of NASH are still under intense investigation, it is well established that Lingguizhugan decoction can suppress the activation of TNF-related immune responses [18]. One interesting finding from our study is that MLGZG suppresses the activation of TLR-4 and TRAF3. The role of TRAF3 in mediating the development of NASH was previously illustrated in an HFD mouse model [7]. Using both HFD- and gene knockout-induced NASH models, TLR-4/TRAF3 activation shifted immune responses and increased insulin resistance, as discussed by Wang [13]. Mitochondrial stress-induced P65 phosphorylation can result in NF-*κ*B activation, leading to I*κ*B degradation and NF-*κ*B nuclear translocation [19]. Researchers found that NF-*κ*B may be involved in an insulin resistance mechanism during hepatocyte exposure to FAs in NASH patients [26]. In this study, phosphorylated P65 upregulation was detected in NASH rats (see Figure 4), while a decrease in phosphorylated P65 was observed after MLGZG feeding. Impairment in TLR4/TRAF3 signaling can cause NF-*κ*B inactivation and subsequently reduce inflammatory responses [27]. These changes played a key role in HFD-induced inflammation in NASH models, which was likely caused by the addition of MLGZG, protecting rats from hepatic injury.

Strikingly, feeding MLGZG significantly reduced the insulin resistance of NASH rats, indicating the pivotal role of modified Lingguizhugan decoction in metabolic disease therapy [16]. In our study, we found that MLGZG markedly decreased TNF-*α* expression (Figure 5). Of note, Ezquerro et al. suggested that TNF-mediated pathways play key roles in inducing apoptosis in hepatocytes [28]. Moreover, other potential modes of action include induction of the inflammatory cytokines IL-1*β* or IL-12 by promoting signaling or induction of a certain subset of T cells. In this report, we showed that MLGZG affects liver inflammatory responses by directly suppressing proinflammatory cytokine activation (Figure 5). Our results are consistent with a scenario in which reduced proinflammatory cytokines play key roles in NASH therapy in rats. A similar trend was detected by ELISA in the HFD cell model (Figure 6). Our results suggest that MLGZG decreases cytokine secretion in HFD-induced NASH livers and that the effect is related to the TLR4/TRAF3 signaling pathway.

It has been reported that Lingguizhugan decoction can be used to treat NASH by regulating lipid genesis [29]. Hepatic TG accumulation is a key step in NASH development. Liu et al. reported that liver lipid accumulation could increase in an HFD-induced NASH model and that the use of Lingguizhugan decoction could significantly reduce lipid uptake [30]. Our study also supports their conclusion; blood TC/TG/LDL increased as the NASH model was established, but after MLGZG treatment, our results indicated that the increases in TC/TG/LDL were significantly reduced. In fact, the expression of SREBP in NASH rats was significantly lower than that in MLGZG-treated rats (see Figure 3). It should be noted that leptin expression was also reduced by MLGZG. The function of leptin is to improve the decomposition of fat; high concentrations of hepatic lipids upregulate this gene in NASH patients. We suggest that MLGZG has a protective role in NASH treatment via suppression of TLR-4-mediated inflammation activation, which subsequently reduces lipid accumulation in the liver. In view of the fact that SREBP and leptin modulate lipid genesis, it is plausible that lipid regulation genes are also involved in the enhanced therapeutic effects of MLGZG.

## 5. Conclusion

Collectively, we have demonstrated that MLGZG shows immunosuppressive and therapeutic effects in treating NASH via the TLR4/TRAF3 signaling pathway. Given the critical role of TLR4/TRAF3 in regulating the immune response, it is conceivable that MLGZG might exhibit improved therapeutic efficacy in treating other metabolic diseases, such as diabetes. With satisfactory inflammation regulatory potential and few side effects, modified Lingguizhugan decoction provides a feasible approach for enhancing therapeutic efficacy in NASH patients. However, the further downstream mechanism of MLGZG treatment still needs to be investigated. Additionally, to develop more inexpensive and safer clinical applications, we need to analyze the active components of MLGZG.

## Figures and Tables

**Table 1:** Primer list.

Target	Sequence	Length
GAPDH	5' AGTGCCAGCCTCGTCTCATA 3′	252 bp
	3' GACTGTGCCGTTGAACTTGC 5′	

SREBP	5' GTTAACGTGGGTCTCCTCCG 3′	168 bp
	3' CCAGCATAGGGGGCATCAAA 5′	

Leptin	5' GGTGGCTGGTTTGTTTCTGT 3′	249 bp
	3' TATGTGGCTGCAGAGGTGAG 5′	

TNF-*α*	5' GATCTCAAAGACAACCAACTG 3′	138 bp
	3' CTGGTATGAAATGGCAAATCG 5′	

IL-1*β*	5' CCTTGTGCAAGTGTCTGAAGC 3′	147 bp
	3' CCCAAGTCAAGGGCTTGGAA 5′	

IL-10	5' CATTATGTGGGCACTGGCCT 3′	156 bp
	3' GTAGATGCCGGGTGGTTCAA 5′	

IL-12	5' CCCATGGATCTTCGGGCAAG 3′	283 bp
	3' TCACGGACCTGCTCACCTAC 5′	

**Table 2:** Antibody list.

Antibody	Cat. no.	Company
TLR-4	AB22048	Abcam
TRAF-3	SC-6933	Santa Cruz
NF-*κ*b p65	8242	Cell Signaling Technology
P-p65	3033	Cell Signaling Technology
*β*-actin (42KD)	4970	Cell Signaling Technology

**Table 3:** Serum lipid analysis of the rat models.

Group	n	TC (*μ*g/L)	TG (*μ*g/L)	HDL (*μ*g/L)	LDL (*μ*g/L)
NORMAL	10	1.254 ± 0.054	1.029 ± 0.090	1.223 ± 0.057	0.390 ± 0.062
HFD	10	1.693 ± 0.052^#^	1.415 ± 0.074^#^	0.772 ± 0.650^#^	0.751 ± 0.065^#^
HFD + MLGZG^L^	10	1.463 ± 0.033^*∗*^	1.307 ± 0.042^*∗*^	0.883 ± 0.082^*∗*^	0.647 ± 0.052^*∗*^
HFD + MLGZG^M^	10	1.340 ± 0.033^*∗*^	1.234 ± 0.060^*∗*^	0.937 ± 0.061^*∗*^	0.589 ± 0.061^*∗*^
HFD + MLGZG^H^	10	1.253 ± 0.041^*∗*^	1.163 ± 0.072^*∗*^	1.105 ± 0.052^*∗*^	0.487 ± 0.050^*∗*^

*Note.* Data are expressed as the mean ± SD. TC: total cholesterol; TG: triglycerides; HDL-C: high-density lipoprotein cholesterol; LDL-C: low-density lipoprotein cholesterol. HFD: high-fat diet; MLGZG^L^: MLGZG low dose; MLGZG^M^: MLGZG moderate dose; MLGZG^H^: MLGZG high dose. # indicates *P* < 0.05, HFD group compared to normal control group; ^*∗*^ indicates *P* < 0.05, MLGZG-treated groups compared to HFD group.

## Data Availability

The data used during the study are available from the corresponding author upon request.

## Abbreviations

NAFLD: Nonalcoholic fatty liver disease
TLRs: Toll-like receptors
TLR-4: Toll-like receptor 4
NLRs: NOD-like receptors
TNF-*α*: Tumor necrosis factor alpha
RIG-I: Retinoic acid-inducible gene I
NF-*κ*B: Nuclear factor kappa B
IRS1: Insulin receptor substrates 1
JNK: C-Jun n-terminal kinase
NASH: Nonalcoholic steatohepatitis
GLUT-2: Glucose transporter type 2
HFD: High-fat diet
TRAF3: TNF receptor-associated factor 3
MLGZG: Modified Lingguizhugan decoction
TNF-R: TNF receptor
SREBP: Sterol-regulatory element binding proteins
TAK-1: Transforming growth factor beta-activated kinase 1.
