# Erratum to “Evaluation of the Mechanism of Modified Lingguizhugan Decoction in the Treatment of Nonalcoholic Fatty Liver Disease”

**DOI:** 10.1155/2022/9782896

**Published:** 2022-08-13

**Authors:** Fuyuan Yang, Jianxiang Zhou, Bo Su, Qiong Zhang, Xuezhi Luo, Fei Wang, Jiangrong Huang, Wei Huang

**Affiliations:** ^1^School of Basic Medicine, Yangtze University Health Science Center, Jingzhou, Hubei 434020, China; ^2^Department of Endocrinology, Jingzhou Hospital of Traditional Chinese Medicine, Third Clinical Medical College, Yangtze University, Jingzhou, Hubei 434020, China; ^3^Hubei College of Chinese Medicine, Jingzhou, Hubei 434020, China

In the article titled “Evaluation of the Mechanism of Modified Lingguizhugan Decoction in the Treatment of Nonalcoholic Fatty Liver Disease” [1], an incorrect author contribution statement was introduced during the production process. The authors' contribution statement should be corrected as follows:

## Authors' Contributions

Fuyuan Yang and Jianxiang Zhou made equal contribution to this work.
